# Poloxamer-Driven Drug Delivery System for Anti-Inflammatory Drugs Using Small-Angle Neutron Scattering Approach

**DOI:** 10.3390/gels11060410

**Published:** 2025-05-30

**Authors:** Rodrigo Rhinow, Margareth K. K. D. Franco, Mont Kumpugdee Vollrath, Guinther Kellermann, Fabiano Yokaichiya

**Affiliations:** 1 Programa de Pós-Graduação em Engenharia e Ciência dos Materiais, Universidade Federal do Paraná, Curitiba 81531-980, Brazil; rodrigo.rhinow@gmail.com (R.R.); keller@fisica.ufpr.br (G.K.); fabiano.yokaichiya@gmail.com (F.Y.); 2 Instituto de Pesquisas Energéticas e Nucleares, São Paulo 05508-000, Brazil; 3 Pharmazeutische Technologie, Berliner Hochschule für Technik, 13353 Berlin, Germany; mont.kumpugdee-vollrath@bht-berlin.de; 4 Department of Physics, Universidade Federal do Paraná, Curitiba 81531-980, Brazil

**Keywords:** SANS, anti-inflammatory drugs, drug delivery carrier

## Abstract

Poloxamer-based drug delivery systems are widely used in the pharmaceutical sector. The structural characterization of these systems is crucial for the development of new drug delivery systems and for the optimization of their properties. In this study, we utilized small-angle neutron scattering (SANS) to investigate the structures of poloxamer-based drug delivery systems. The samples were measured using the SANS technique on the VSANS-V16 instrument at Helmholtz-Zentrum Berlin (HZB), Germany. The samples contained 20% poloxamer (P407) and 0.2% of a drug (ibuprofen, ketoprofen, diclofenac) in deuterated water (D_2_O) for SANS. The samples varied in terms of temperature analysis (25 °C, common storage temperature; 37 °C, human body temperature; 40 °C, fever temperature). The data analysis involved modeling the data using a Python-based routine. The model used consisted of an isotropic solution of polydisperse spherical micelles. The intensity as a function of the scattering vector was modeled as the product of the form factor and the interparticle structure factor, with the latter described within the local monodisperse approximation regime. Additionally, a scattering contribution was observed, which was associated with the presence of crystalline superstructures formed by micelles that organized into a cubic structure. The data analysis provided important information about the system, such as the average radius, the size distribution, and the thickness of the layer surrounding the micellar core. The results will contribute to the development and optimization of new drug delivery systems that are more effective and safer for medical applications.

## 1. Introduction

Poloxamer-based drug carrier systems have emerged as a promising approach for sustained drug release due to their properties, such as their low toxicity, physicochemical characteristics, amphiphilicity, and ability to form micelles in aqueous solutions [1]. Poloxamers, also known as pluronics, which organize themselves into micellar aggregates, are a class of amphiphilic block copolymers that are widely used as drug delivery systems due to their unique physicochemical properties [2]. These copolymers consist of hydrophilic poly(ethylene oxide) (PEO) blocks and hydrophobic poly(propylene oxide) (PPO) blocks, which enable them to self-assemble into micelles in aqueous solutions [3]. The micellar structures formed by poloxamers can effectively encapsulate hydrophobic drugs, improving their solubility, stability, and bioavailability [4]. Additionally, poloxamers can modulate the release rate of the encapsulated drug, providing controlled or sustained drug release, which is crucial in minimizing side effects and enhancing the therapeutic efficacy [5]. Their versatility allows poloxamers to be used in various drug delivery applications, including oral, parenteral, and topical routes [6]. Poloxamer-based formulations also exhibit excellent biocompatibility and low toxicity, making them attractive for use in both the pharmaceutical and biomedical fields [4]. Furthermore, the ability to tailor their compositions and properties enables the design of drug delivery systems for a wide range of therapeutic agents, including anti-inflammatory drugs, anticancer agents, and antibiotics [7].

Ibuprofen, ketoprofen, and diclofenac are all nonsteroidal anti-inflammatory drugs (NSAIDs) used to treat pain, inflammation, and fever, but they differ in terms of their chemical structures, potency, and side effects [8,9]. These drugs work by inhibiting cyclooxygenase enzymes and subsequently reduce the synthesis of prostaglandin chemicals [10,11] involved in inflammation, pain, and fever. They are effective for a variety of conditions, including headaches, muscle aches, menstrual cramps, arthritis, and minor injuries [12,13,14,15]. Despite their widespread use, it is important to follow suitable dosing guidelines, as their excessive use can lead to side effects such as gastrointestinal issues when taken orally, kidney damage, and an increased risk of heart problems [16,17,18,19,20,21,22]. Moreover, these drugs are also known for their poor solubility [23,24,25]. In order to address these challenges, various drug delivery systems, such as nanoparticles, liposomes, and solid–lipid nanoparticles, are employed to improve the solubility, bioavailability, and controlled release of these drugs [26]. These carriers can protect the drug from degradation, provide sustained release, and reduce the frequency of dosing [27], ultimately improving patient compliance. Additionally, by targeting specific tissue types or organs, drug carriers can help to minimize the systemic side effects commonly associated with these drugs, such as stomach ulcers and gastrointestinal bleeding [28,29], where diclofenac is more aggressive [17].

The use of small-angle neutron scattering (SANS) plays a significant role in studying drug carriers, particularly in the characterization of their structural properties at the nanoscale [30]. SANS allows for the investigation of the size, shape, and distribution of drug delivery systems such as liposomes, micelles, and nanoparticles, providing insights into their formation, stability, and interactions with biological environments [31]. This technique allows us to obtain valuable information about the morphology and internal organization of these carriers, which is crucial in optimizing drug release profiles, improving bioavailability, and ensuring targeted delivery [32]. The ability to probe these structures in a non-destructive manner makes SANS a powerful tool in the development of more effective and efficient pharmaceutical carriers.

The use of drug carriers for ibuprofen, ketoprofen, and diclofenac delivery has gained significant attention in pharmaceutical research, as these carriers can enhance the drug’s therapeutic efficacy and reduce side effects [29]. Thus, in this study, we used the SANS technique to investigate how the anti-inflammatory drugs ibuprofen, ketoprofen, and diclofenac affect the structures of poloxamer-based (P407) drug carrier systems.

## 2. Results and Discussion

### 2.1. Results

To perform accurate data modeling, a computational model was developed in Python 3.12 using libraries such as numpy, matplotlib, and scipy [33,34,35]. The fitting process was performed using the least squares method, available in the scipy library, which minimizes the differences between the calculated curve and the experimental curve. In this way, the program can determine the ideal parameters so that the computational model coherently resembles the experimental curves.

For small-angle scattering (SAS), the intensity function relates the intensity of the measured scattering to the scattering vector (*q*). The intensity function can be decomposed into two main components: the form factor (*P*(*q*)) and the structure factor (*S*(*q*)). The form factor describes the contribution to the scattering of a single particle or structure within the sample. It is determined by the Fourier transform of the scattering density of the particle, providing information about the size, shape, and internal distribution of the particle. The structure factor considers the interactions between particles in the sample. It reflects the pattern of spatial correlations between particles and can reveal information about the nanoscopic organization or the formation of aggregates in the sample. Additional factors can be included to describe other parameters, such as the polydispersity of the sample and the semicrystalline contribution, as in the present study.

#### 2.1.1. Form Factor

The form factor is a function that describes the scattering of neutrons by a specific particle or structure, taking into account its shape and distribution. This function is essential to elucidate the scattering patterns obtained and to extract detailed information about the size, shape, and orientation of particles in a solution. Three form factors were tested for data modeling: a uniform homogeneous sphere, a core–shell model, and a spherical core with attached Gaussian chains. The normalized form factor of a homogeneous sphere is given by

(1) $$P_{sphere}\left(q,R_{s}\right)=\left[\frac{3J_{1}\left(qR_{s}\right)}{qR_{s}}\right]={\left[\frac{3}{{qR}_{s}}\left(\frac{sin\left({qR}_{s}\right)}{{\left(qR_{s}\right)}^{2}}-\frac{cos\left({qR}_{s}\right)}{qR_{s}}\right)\right]}^{2}=\frac{9{\left[sin\left(qR_{s}\right)-qR_{s}cos\left(qR_{s}\right)\right]}^{2}}{{\left(qR_{s}\right)}^{6}}$$

where *R_s_* represents the radius of the hydrophobic core of the micelle, and *J*_1_ is the first spherical Bessel function, given by $J_{1}\left(x\right)=\left(\frac{sin\left(x\right)}{x^{2}}-\frac{cos\left(x\right)}{x}\right)$. Other form factors were considered, such as the core–shell model (Equation (3)):

$$F_{Core-Shell}\left(q\right)=3\left[\frac{V_{core}\left(\rho_{core}-\rho_{solvent}\right)J_{1}\left(qR_{core}\right)}{qR_{core}}+\frac{V_{shell}\left(\rho_{shell}-\rho_{solvent}\right)J_{1}\left(qR_{shell}\right)}{qR_{shell}}\right]=$$

(2) $$=\frac{3}{V_{shell}}\left[V_{core}\left(\rho_{core}-\rho_{solvent}\right)\frac{\sin\left(qR_{core}\right)-qR_{core}\cos\left(qR_{core}\right)}{{\left(qR_{core}\right)}^{3}}+V_{shell}\left(\rho_{shell}-\rho_{solvent}\right)\frac{\sin\left(qR_{shell}\right)-qR_{shell}\cos\left(qR_{shell}\right)}{{\left(qR_{shell}\right)}^{3}}\right]$$

The subscripts shell, core, and solvent refer to the shell, the core of the micelles, and the solvent in which the sample is embedded, respectively. Moreover, *ρ* represents the scattering length density, *V_core_* is the core volume, and *V_shell_* is the total volume of the nanoparticle. Using Equation (2), we can express the form factor of the core–shell model (Equation (3)), obtaining the form factor of this model:

(3) $$P_{Core-Shell}\left(q\right)=\frac{k}{V_{s}}F_{Core-Shell}^{2}$$

The model of a spherical, homogeneous micellar core with attached Gaussian chains is given by the following form factor (Equation (4)):

(4) $$P\left(q,R_{s}\right)=N^{2}\gamma_{s}^{2}P_{s}\left(q,R_{s}\right)+N\gamma_{c}^{2}P_{c}\left(q,R_{g}\right)+N\left(N-1\right)\gamma_{c}^{2}S_{cc}+2N^{2}\gamma_{c}\gamma_{s}S_{sc}$$

where the subscripts s and c refer to the core of uniform spherical micelles (core radius = *R_s_*) and the attached Gaussian chains (radius of gyration = *R_g_*), and *N* is the aggregation number of the micelle. The total excess scattering density of a chain within the core is given by *γ_s_*, or, within the corona, by *γ_c_*. The term *P_s_*(*q*, *R_s_*) is the normalized autocorrelation term for a uniform sphere, and *P_c_*(*q*, *R_g_*) is the autocorrelation term for Gaussian chains. *S_sc_* is the cross-interference term between the sphere and the Gaussian chain starting at the sphere’s surface, and *S_cc_* is the interference term between Gaussian chains attached to the surface of a sphere.

Both models—the core–shell and the spherical core with Gaussian chains (Equations (3) and (4))—incorporate a larger number of parameters in the data modeling as compared to the homogeneous sphere model (Equation (1)). However, we believe that the difference (scattering length) between water and the micellar chains external to the core is not significant, meaning that the scattering lengths of the water and micellar chains have similar values. Thus, the interference factor (*S*(*q*)), together with the contribution of the ordered structure, exerts a more significant influence on the curve shape compared to the form factor. To optimize the simulation time, the form factor equation for a homogeneous sphere, as described in Equation (1), was selected.

#### 2.1.2. Structure Factor

The structure factor in X-ray scattering is an essential quantity in the characterization of high-concentration structures, as shown in this study, with poloxamer concentrations higher than 20%. This parameter is directly related to the distribution of relative distances between micelles. Considering that the particle concentration is nonzero, the scattering cross-section includes contributions from the interparticle structure factor *S*(*q*), which becomes more relevant as the concentration increases. To describe the behavior of the particle concentration, an interference function based on the solution of the Ornstein–Zernike integral equation is used, which establishes a relationship between the direct correlation function *c*(*r*) and the total correlation function *h*(*r*). Here, $\bar{N}=N/V$ represents the number density of particles.

(5) $$h\left(\vec{r}\right)=c\left(\vec{r}\right)+\bar{N}\int d{\vec{r}}^{\prime }c\left(\vec{r}-{\vec{r}}^{\prime }\right)h({\vec{r}}^{\prime })$$

Jerome K. Percus and George J. Yevick proposed a closure relation for systems of rigid spherical particles, as a simple model of liquids, known as the Percus–Yevick approximation [36,37]. This approximation provides an approximate relation between the total correlation functions $h\left(\vec{r}\right)$ and the direct correlation functions $c\left(\vec{r}\right)$, given by

(6) $$c\left(r\right)=\left(h\left(r\right)+1\right)\left(1-e^{V\left(r\right)\beta }\right)$$

where *β* = −1/*k_b_T*, with *k_b_* being the Boltzmann constant, *T* the temperature, and *V*(*r*) the interaction potential of hard spheres between the particles, such that

*V*(*r*) = 0 for *r* > *D* (7)

*V*(*r*) = *∞* for *r* < *D* (8)

where *r* is the effective interaction radius and *D* is the sphere diameter. Since the potential *V*(*r*) is either zero or infinite, the Boltzmann factor *e^V^*^(*r*)*β*^ is equal to one or zero, regardless of the temperature *T*. Therefore, the structure of a hard-sphere fluid is independent of the temperature. This leaves only two parameters to be analyzed: the radius of the rigid part R and the volume fraction *ϕ*(*R_eff_*), resulting in Equation (14).

(9) $$\delta =\frac{{\left(1+2\phi \right)}^{2}}{{\left(1-\phi \right)}^{4}}$$

(10) $$\beta =\frac{-3\phi {\left(2+\phi \right)}^{2}}{2{\left(1-\phi \right)}^{4}}$$

(11) $$f_{1}\left(Q\right)=\frac{sin\left(Q\right)-Qcos\left(Q\right)}{Q^{3}}$$

(12) $$f_{2}\left(Q\right)=\frac{2Qsin\left(Q\right)-\left(Q^{2}-2\right)cos\left(Q\right)-2}{Q^{4}}$$

(13) $$f_{3}\left(Q\right)=\frac{\left(4Q^{3}-24Q\right)sin\left(Q\right)-\left(Q^{4}-12Q^{2}+24\right)cos\left(Q\right)+24}{Q^{6}}$$

(14) $$S_{PY}\left(Q,R_{eff}\right)=\frac{1}{1+24\phi \left(\delta f_{1}+\beta f_{2}+\frac{1}{2}\delta \phi f_{3}\right)}$$

where *Q* = 2*qR_eff_*, *ϕ* = *ϕ*(*R_eff_*), and *R_eff_* is the effective interaction radius. The interference equation is used in a product with the sphere form factor to describe the intensity detected in concentrated systems [38,39,40].

#### 2.1.3. Polydispersity

A Gaussian function (Equation (15)) is also used to simulate the polydispersity of the micelles [41], for both the first population (micelles) and the second population (free polymer chains):

(15) $$f\left(R_{s}\right)=\frac{1}{\sigma \sqrt{2\pi }}exp\left(\frac{-{\left(R_{s}-R_{m}\right)}^{2}}{2\sigma^{2}}\right)$$

where *R_m_* is the average radius of the particles, and *σ* is the standard deviation of the micelles.

#### 2.1.4. Ordered Contribution

The form factors, structure factors, and Gaussian polydispersity function were not sufficient to fit the model to the experimental data, which could be attributed to the formation of cubic superstructures, as observed in SANS measurements and reported in the literature [42,43].

Since the relative position of the peaks with respect to the scattering vector (*q*/*q*_0_) is known, we observe that the SANS intensity spectrum presents the contribution of a face-centered cubic (FCC) structure. Thus, it is possible to obtain the structural parameters of the sample, since the position of the peaks is inversely related to the distances between the scattering planes in the material, with d being the lattice parameter, as described in Equation (16):

(16) $$d=\frac{2\pi }{q}$$

Consequently, a model was developed that takes into account the interparticle interaction of the superstructure, modeling the characteristic curves at the positions of the known peaks (Table 1).

In order to consider the contributions of the superstructure, a convolution of Gaussian functions at the known positions was added:

(17) $$g_{q_{0}/q}\left(q\right)=\frac{1}{\sigma_{FCC}\sqrt{2\pi }}exp\left(\frac{-{\left(q-q_{0}\right)}^{2}}{2\sigma_{FCC}^{2}}\right)$$

where *q*_0_ is the peak position and *σ_FCC_* is the standard deviation of the function. Thus, the structural contribution for an *FCC* structure is given by Equation (18):

(18) $$I_{FCC}\left(q\right)=k_{3}(a_{1}g_{\sqrt{1}}\left(q\right)+a_{2}g_{\sqrt{\frac{4}{3}}}\left(q\right)+a_{3}g_{\sqrt{\frac{8}{3}}}\left(q\right)+a_{4}g_{\sqrt{\frac{11}{3}}}\left(q\right)+a_{5}g_{\sqrt{4}}\left(q\right))$$

where *a*_1,…,5_ are the characteristic amplitudes of the structure’s peaks.

The SANS technique reveals intensity curves for the poloxamer P407 samples that resemble models of polydisperse spherical micelles [41], which is in agreement with the literature [44,45]. The model is given by the local monodisperse approximation (LMA), described by Pedersen and Gerstenberg [46,47,48], taking into account both the form factor and the interparticle interference factor. A Gaussian function was used to describe the radial distribution of the micelles, together with a contribution from the observed cubic superstructure [41].

A new contribution was added, arising from a second population of nanoparticles that were smaller than micelles, which we believe to be polymer chains that did not form micelles. However, this population had a much smaller concentration compared to the first population, and it was not necessary to add the contribution of a structure factor. Therefore, our model to describe drug carrier systems is given by the following Equation (19):

(19) $$I\left(q\right)=\sum_{i=1}^{l}\left[{k_{1}V}_{1}^{2}\left(R_{1s,i}\right)P_{1}\left(q,R_{1s,i}\right)f_{1}\left(R_{1s,i}\right)S_{PY}\left(q,R_{1eff}\right)\Delta R_{1s,i}+\;k_{2}V_{2}^{2}\left(R_{2s,i}\right)P_{2}\left(q,R_{2s,i}\right)f_{2}\left(R_{2s,i}\right)\Delta R_{2s,i}\right]+I_{FCC}\left(q\right)+bkg$$

where indices 1 and 2 represent the first and second particle populations, respectively; *k* is the scaling factor of their respective contributions to the obtained intensity curve; and *bkg* is the background scattering, a constant value that does not result from the structures and nanoparticles (micelles and polymer chains) present in the sample (background). For a good approximation, l = 400 was chosen. With this model, it was possible to achieve a precise fit of the obtained curves.

#### 2.1.5. Porod Constant

In the interest of removing the portion of the scattering corresponding to a constant contribution to the scattering intensity, Porod’s Law is used [36], which states that, in the high *q* region, the intensity is proportional to the constant *K_p_*, known as Porod’s constant.

(20) $$I\left(q\right)=\frac{K_{p}}{q^{4}}+bkg\;\mathrm{for}\;q\to \infty$$

**Figure 1:**
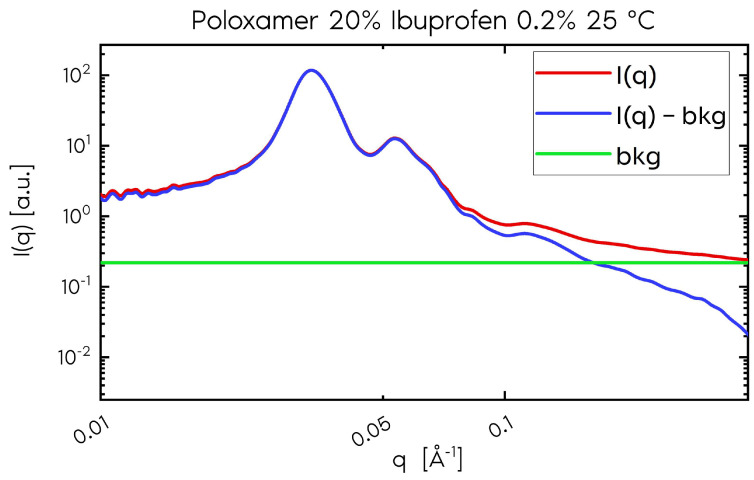
Scattering intensity of the P407 sample (20%) and ibuprofen (0.2%) at 25 °C. The red curve represents the total scattering, and the blue curve represents the scattering without the constant contribution, depicted by the green curve. We observe that the difference in contribution is only significant for *q* > 0.075 Å^−1^.

By multiplying Equation (20) by *q*^4^, we obtain Equation (21):

(21) $$I\left(q\right)q^{4}=bkgq^{4}+K_{p}$$

which describes linear behavior for high *q* values, allowing the determination of the slope (*bkg*), which represents a constant contribution from variations in the electron density at the interface between the nanoparticles and their surrounding medium. We can then subtract *bkg* from the total intensity value and obtain exclusively the scattering contribution from the nanoparticles (Figure 1).

### 2.2. Discussion

**Figure 2:**
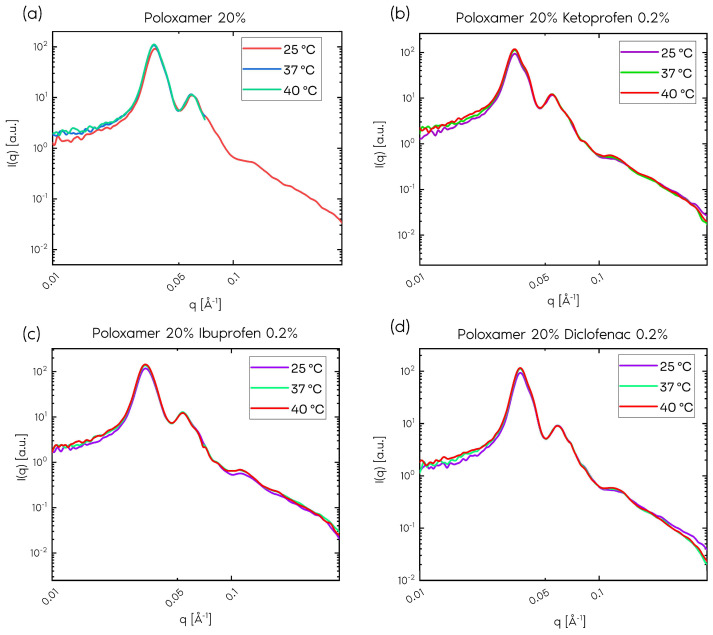
Small-angle neutron scattering (SANS) curves for sample with 20% poloxamer (**a**), 20% poloxamer and 0.2% ketoprofen (**b**), 20% poloxamer and 0.2% ibuprofen (**c**), and 20% poloxamer and 0.2% diclofenac (**d**).

No significant difference was observed in the scattering profiles in relation to the temperature in the control sample (without any drug) (Figure 2a). Figure 2 shows the results obtained in the presence of drugs. For the curves shown in Figure 2, no significant change was observed in relation to the temperature, following the behavior of the control sample.

**Figure 3:**
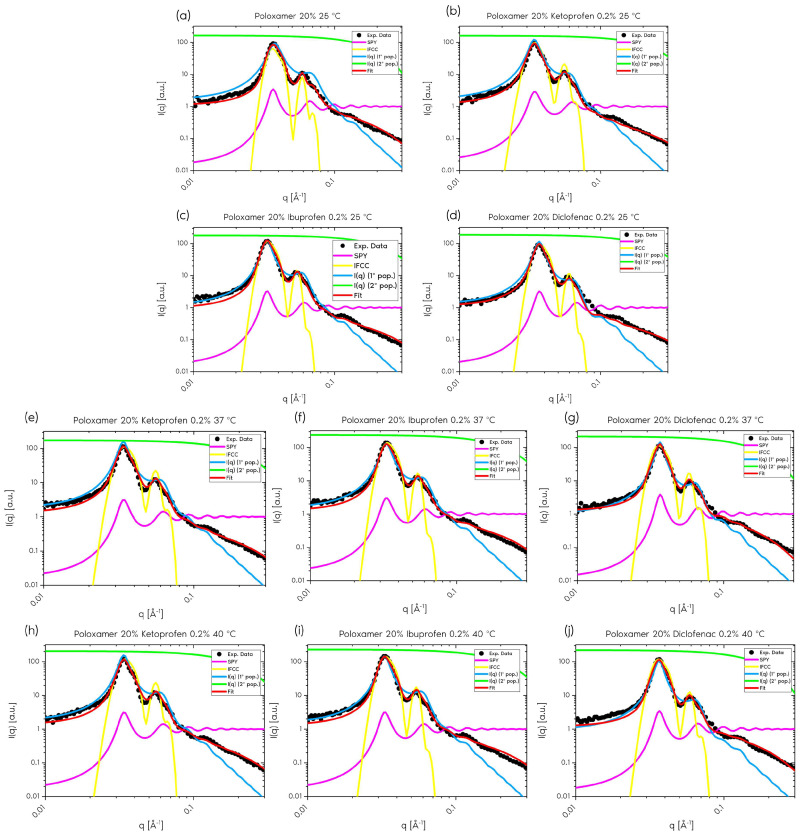
Small-angle neutron scattering curves of (**a**) P407 20% without drug addition (25 °C) and with (**b**,**e**,**h**) ketoprofen 0.2%, (**c**,**f**,**i**) ibuprofen 0.2%, and (**d**,**g**,**j**) diclofenac 0.2%, at (**a**–**d**) 25 °C, (**e**–**g**) 37 °C, and (**h**–**j**) 40 °C. The functions used to model the data are multiplied by a scaling factor for better visualization.

For this study, analyses were performed with the anti-inflammatory drugs ibuprofen, ketoprofen, and diclofenac. Figure 3 shows the scattering curves and the functions used for data modeling, for P407 samples without and with ibuprofen, ketoprofen, and diclofenac at 25 °C, 37 °C, and 40 °C.

**Figure 4:**
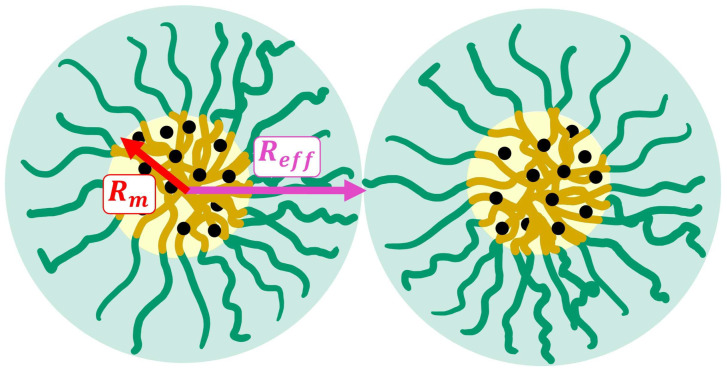
Schematic representing the average radius of the micelle’s core (*R_m_*) and the effective interaction radius between micelles (*R_eff_*).

**Figure 5:**
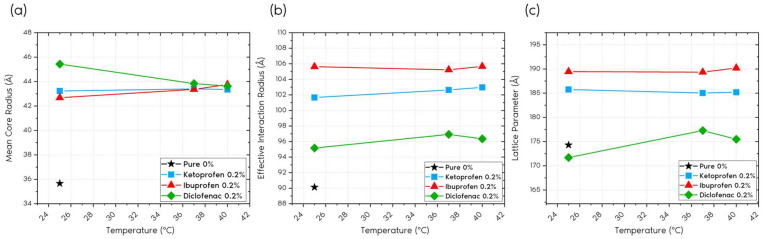
(**a**) Variation in the average radius parameter (*R_m_*) of the micelle core with temperature; (**b**) variation in the effective radius (*R_eff_*) with temperature; and (**c**) variation in the lattice parameter (Equation (16)) with temperature for P407 samples containing ketoprofen, ibuprofen, and diclofenac.

Table 2 and Table 3 list the data of the parameters used to model the scattering curves of the nanoparticles and the superstructures formed by P407, with which the mean and effective radii (Figure 4) of the micelles were estimated. Figure 5 shows the behavior of these parameters in relation to the temperature for each of the systems.

Each sample presented different behavior as a function of the temperature. The slight growth of the core was observed, as in the case of ibuprofen.

A slight temperature-dependent decrease was observed for diclofenac, with a value of 1.81 Å. We can observe that the average radius of the interior of the micelle, *R_m_*, does not vary as a function of the temperature (Figure 5a).

The graphs in Figure 5b show that the effective radius (*R_eff_*) is not significantly affected by the temperature; however, small differences are observed in this parameter according to the drug incorporated in P407.

Another important parameter analyzed was the superstructure lattice parameter, obtained by Equation (16). When observing the graphs shown in Figure 5c, no direct correlation was identified between the temperature and lattice parameter. However, samples containing drugs, such as diclofenac 0.2%, presented similar lattice parameters, albeit slightly smaller than that of the control sample, without the inclusion of drugs.

The polydispersity of the micelle interior, *σ*, ranged from 9 to 13 Å for all samples. The second population of nanoparticles (polymer chains) presented an average radius of approximately 15% of the total micelle radius, with a variation (*σ*_2_) observed between 1 and 3 Å. The volume fraction (*ϕ*) presented values between 0.41 and 0.53, varying from measurement to measurement.

## 3. Conclusions

The presented model proved to be effective in modeling small-angle neutron scattering (SANS) data for the studied anti-inflammatory drugs, allowing the investigation of the relationship between the structural characteristics of the samples with and without drugs as a function of the temperature. No significant variations were observed in the mean radius of the interior of the micelles, in the effective interaction radius, or in the lattice parameter with the variation in temperature. Some discrepant values may have been due to the incorporated drug and its characteristics, such as its polarity and size, for example.

Obtaining structural parameters through data modeling and studying the phase transitions of drug carrier systems in detail can bring significant benefits to the pharmaceutical industry. These parameters, such as the mean micelle radius, the effective interaction radius, and the lattice parameter, provide crucial information about the organization and behavior of micelles under different temperature and concentration conditions.

This knowledge can be used to optimize drug formulations, improving the stability, solubility, and bioavailability of encapsulated drugs and, most importantly, expanding the possible applications. This can result in greater therapeutic efficiency and a better patient experience, driving innovation in new drugs and their forms of administration.

## 4. Materials and Methods

### 4.1. Samples

**Figure 6:**
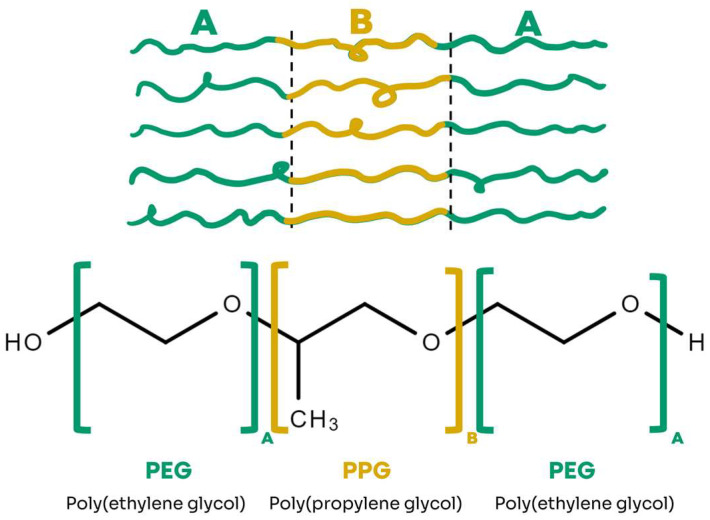
Chemical structure of the ABA-type triblock copolymer, Poloxamer (P407).

The samples analyzed at HZB using the SANS technique contained 20% poloxamer (P407). P407 is a tri-block copolymer composed of a hydrophobic central block of poly(propylene glycol) (PPG), surrounded by two hydrophilic blocks of poly(ethylene glycol) (PEG). The central block consists of 56 PPG units, represented by B, while the ends are formed by 101 PEG units (A), as illustrated in Figure 6. P407 has a molecular mass of 12,600 g/mol and a hydrophilic–lipophilic balance (HLB) of 22 [49].

**Figure 7:**
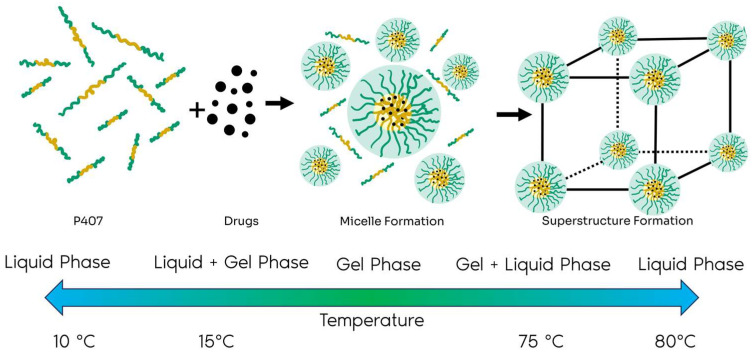
Schematic illustration of the formation of micelles and gels of P407. The phase transitions include liquid–gel, which occurs at approximately 15 °C, and gel–liquid, observed at around 75 °C. Adapted from [50].

P407 samples for SANS measurements were prepared with 0.2% concentrations of the anti-inflammatory drugs ibuprofen, ketoprofen, and diclofenac in deuterated water (D_2_O). The formation of micelles, encapsulating the drugs inside, is expected when the P407 concentration is sufficiently high, above its CMC, and with supramicellar aggregates, as shown in Figure 7. The samples varied in their incubation temperatures: 25 °C (room temperature), 37 °C (human body temperature), and 40 °C (hyperthermia).

To prepare the samples, the transition from liquid to the lower gel phase, which occurs at temperatures below 15 °C, as proposed by Schmolka in 1972 [51], was respected. P407 was slowly added to a container with water in an inverted water bath with ice on a magnetic stirrer until completely dissolved. This was followed by an equilibration period of 8 to 24 h in a refrigerator. The inverted water bath was necessary to maintain the sample in its liquid state, allowing the incorporation of the drug.

### 4.2. Experimental Setup

**Figure 8:**
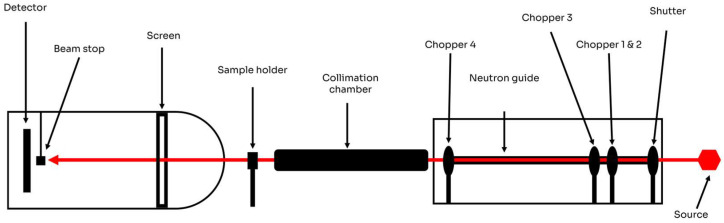
V16/VSANS equipment from chopper section (**left** side) to detector chamber (**right** side).

Small-Angle Neutron Scattering. Neutron scattering measurements (SANS) were performed with a VSANS-V16 time-of-flight (very small-angle scattering) instrument at Helmholtz-Zentrum Berlin, Germany (Figure 8).

Two configurations were used based on the distance from the detector to the sample: 1.7 m with neutron wavelengths of 1.8–3.8 Å and 11 m with neutron wavelengths of 1.6–9.2 Å. The samples were placed in Hellma 110 QS cuvettes, and a waiting period of 30 min between acquisitions was used to ensure temperature stabilization. The samples were prepared in D_2_O to ensure significant contrast between the solvent and the nanoparticles, and they were measured at 25, 37, and 40 °C, as shown in Table 4. Corrections for sample transmission, background detector counts, empty cell scattering, and detector efficiency were included in the final data reduction. The SANS data were radially averaged and merged to give a total *q* range of 0.005–0.5 Å

## Figures and Tables

**Table 1:** Relative positions of diffraction peaks for BCC and FCC cubic structures.

$\mathrm{Relative}\;\mathrm{Position}\;(q/q_{0})$	
BCC	FCC
$\sqrt{1},\sqrt{2},\sqrt{3},\sqrt{4},\sqrt{5}$	$\sqrt{1},\sqrt{\frac{4}{3}},\sqrt{\frac{8}{3}},\sqrt{\frac{11}{3}},\sqrt{4}$

**Table 2:** Parameters used for the modeling of nanoparticles (micelles and polymer chains) in the scattering function.

Name	Concentration (%)	Temperature (°C)	R_m_ (Å)	$\sigma_{1}$	R_m2_ (Å)	$\sigma_{2}$	bkg
Pure	0	25	35.66	8.20	10.30	1.38	0.031
Ketoprofen	0.2	25	43.23	9.87	6.32	1.67	0.016
		37	43.40	10.82	7.93	1.79	0.017
		40	43.35	10.81	7.53	2.25	0.017
Ibuprofen		25	42.68	9.50	7.17	1.78	0.017
		37	43.36	9.60	7.65	1.80	0.017
		40	43.76	9.88	7.60	1.90	0.018
Diclofenac		25	45.43	8.33	6.44	2.01	0.033
		37	43.83	9.52	8.87	2.69	0.010
		40	43.62	8.41	10.23	1.89	0.024

**Table 3:** Parameters used for the modeling of the superstructure in the scattering function.

Name	Lattice Parameter (Å)	R_eff_ (Å)	ϕ	$\sigma_{FCC}$	$a_{1}$	$a_{2}$	$a_{3}$	$a_{4}$	$a_{5}$
Pure	174.29	90.12	0.46	0.0025	10.12	3.97	1.36	0.08	0.04
Ketoprofen	185.76	101.66	0.48	0.0030	9.68	2.00	2.22	0.08	0.08
	185.05	102.63	0.50	0.0030	9.83	4.00	2.15	0.08	0.10
	185.18	102.97	0.50	0.0029	9.83	3.96	2.13	0.07	0.09
Ibuprofen	189.46	105.63	0.50	0.0026	10	4.00	1.30	0.01	0.01
	189.34	105.23	0.49	0.0026	10	4.00	1.30	0.01	0.01
	190.20	105.66	0.50	0.0026	10	4.00	1.30	0.01	0.01
Diclofenac	171.70	95.17	0.50	0.0029	10	4.00	1.30	0.01	0.01
	177.28	96.92	0.53	0.0028	10.48	4.50	1.70	0.12	0.05
	175.47	96.35	0.51	0.0029	10.32	4.17	1.19	0.03	0.03

**Table 4:** Table of neutron low-angle scattering (SANS) measurements performed using the VSANS-16 instrument, HZB.

Drug	Concentration (%)	Temperature (°C)
N/A	0	25
Diclofenac	0.2	25, 37, 40
Ketoprofen	0.2	25, 37, 40
Ibuprofen	0.2	25, 37, 40

## Data Availability

The data presented in this study are available on request from the corresponding author.
